# Can Spatiotemporal Fluoride (^18^F^−^) Uptake be Used to Assess Bone Formation in the Tibia? A Longitudinal Study Using PET/CT

**DOI:** 10.1007/s11999-017-5250-8

**Published:** 2017-02-01

**Authors:** Henrik Lundblad, Charlotte Karlsson-Thur, Gerald Q. Maguire, Cathrine Jonsson, Marilyn E. Noz, Michael P. Zeleznik, Lars Weidenhielm

**Affiliations:** 1 Department of Molecular Medicine and Surgery, Karolinska Institute, Stockholm, Sweden; 2 School of Information and Communication Technology, KTH Royal Institute of Technology, Stockholm, Sweden; 3 Department of Medical Physics, Karolinska University Hospital Solna, Stockholm, Sweden; 4 Department of Radiology, New York University, New York, NY USA; 5 School of Computing, College of Engineering, University of Utah, Salt Lake City, UT USA; 6 Department of Molecular Medicine and Surgery, K l, Orthopedics, A2:07, 171 76 Stockholm, Sweden

## Abstract

**Background:**

When a bone is broken for any reason, it is important for the orthopaedic surgeon to know how bone healing is progressing. There has been resurgence in the use of the fluoride (^18^F^−^) ion to evaluate various bone conditions. This has been made possible by availability of positron emission tomography (PET)/CT hybrid scanners together with cyclotrons. Absorbed on the bone surface from blood flow, ^18^F^−^ attaches to the osteoblasts in cancellous bone and acts as a pharmacokinetic agent, which reflects the local physiologic activity of bone. This is important because it shows bone formation indicating that the bone is healing or no bone formation indicating no healing. As ^18^F^−^ is extracted from blood in proportion to blood flow and bone formation, it thus enables determination of bone healing progress.

**Questions/purposes:**

The primary objective of this study was to determine whether videos showing the spatiotemporal uptake of ^18^F^−^ via PET bone scans could show problematic bone healing in patients with complex tibia conditions. A secondary objective was to determine if semiquantification of radionuclide uptake was consistent with bone healing.

**Methods:**

This study investigated measurements of tibia bone formation in patients with complex fractures, osteomyelitis, and osteotomies treated with a Taylor Spatial Frame^TM^ (TSF) by comparing clinical healing progress with spatiotemporal fluoride (^18^F^−^) uptake and the semiquantitative standardized uptake value (SUV). This procedure included static and dynamic image acquisition. For intrapatient volumes acquired at different times, the CT and PET data were spatially registered to bring the ends of the bones that were supposed to heal into alignment. To qualitatively observe how and where bone formation was occurring, time-sequenced volumes were reconstructed and viewed as a video. To semiquantify the uptake, the mean and maximum SUVs (SUVmean, SUVmax) were calculated for the ends of the bones that were supposed to heal and for normal bone, using a spherical volume of interest drawn on the registered volumes. To make the semiquantitative data comparable for all patients with multiple examinations, the SUVmean and SUVmax difference per day (SUVmeanDPD and SUVmaxDPD) between the first PET/CT scan and each subsequent one was calculated. Indicators of poor healing progress were (1) uneven distribution of the radionuclide uptake between ends of the bones that were supposed to heal as seen in the video or, (2) low absolute magnitude of the SUV difference data. Twenty-four patients treated between October 2013 and April 2015 with a TSF gave informed consent to be examined with ^18^F^−^ PET/CT bone scans. Twenty-two patients successfully completed treatment, one of whom had only one PET/CT scan.

**Results:**

Observation of ^18^F^−^ uptake was able to identify three patients whose healing progress was poor, indicated by uneven distribution of radionuclide uptake across the ends of the bones that were supposed to heal. An absolute magnitude of the SUVmaxDPD of 0.18 or greater indicated good bone formation progress. This was verified in 10 patients by the days between the operation to attach and to remove the TSF being less than 250 days, whereas other SUVmaxDPD values were ambiguous, with 11 patients achieving successful completion.

**Conclusions:**

Observation of the spatiotemporal uptake of ^18^F^−^ appears to be a promising method to enable the clinician to assess the progress of bone formation in different parts of the bone. Bone uptake which is uneven across the ends of bone that were supposed to heal or very low bone uptake might indicate impaired bone healing where early intervention may then be needed. However, semiquantification of ^18^F^−^ uptake (SUVmaxDPD), SUVmeanDPD) was ambiguous in showing consistency with the bone-healing progress.

**Level of Evidence:**

Level III, diagnostic study.

**Electronic supplementary material:**

The online version of this article (doi:10.1007/s11999-017-5250-8) contains supplementary material, which is available to authorized users.

## Introduction

The ready availability of positron emission tomography (PET)/CT hybrid scanners together with cyclotrons has led to a resurgence in the use of the fluoride (^18^F^−^) ion to evaluate various bone conditions [11, 13, 16]. Compounded as a salt (sodium [^18^F^−^] fluoride), ^18^F^−^ is rapidly taken up by bone, particularly healing bone, thus giving a high bone-to-background contrast [7], making it an excellent bone-imaging agent. The ^18^F^−^ ion is incorporated in the hydroxyapatite crystal of bone (Ca_10_[{(PO}_4_]_6_[OH]_2_), reflecting the osteoblastic activity of the bone by replacing the hydroxyl groups to form fluoroapatite (Ca_10_[{PO}_4_]_6_F_2_) [6, 22]. It was confirmed by animal [19, 27] and crystal and mineral [3, 8, 18] studies that ^18^F^−^ is directly incorporated into the apatite lattice, reducing the solubility of bone. Absorbed onto the bone surface from blood flow, ^18^F^−^ attaches to the osteoblasts in cancellous bone and acts as a pharmacokinetic agent, which reflects the activity of bone and also describes blood perfusion [10, 43]. The amount of ^18^F^−^ used in diagnostic procedures, such as used here, is orders of magnitude less than the smallest amount known to have a therapeutic effect.

The Taylor Spatial Frame^TM^ (TSF) (Smith & Nephew, Memphis, TN, USA) is a circular frame used as an external fixator to treat complex fractures, nonunions, and other bony abnormalities [23, 39]. In some patients absent or delayed callus formation in the fracture site or distraction gap may occur. This could lead to a prolonged time until the bone is completely healed and the fixator can be removed, which is problematic as it can lead to significant morbidities. The patient will be at increased risk of psychological stress, pin-tract infections, persistent pain, and osteopenia. Several techniques to manage poor callus formation have been described from systemic administration of pharmaceutical agents such as bisphosphonates, local exogenous administration of growth factors such as BMPs and bone marrow cells, to the use of externally applied low-intensity pulsed ultrasound and pulsed electromagnetic fields [3, 7–10]. Another technique is the so-called accordion maneuver [28], where the mechanical effect of alternatively applied compression and distraction forces are used to stimulate bone formation. This principle is based on the intrinsic capacity of the bone to regenerate under a controlled mechanical environment [2, 29]. If, early during the treatment, it were possible to identify patients with a high risk of delayed or nonunion, this could identify patients who might benefit from early intervention to optimize treatment. Currently, planar radiography and CT in combination with clinical examinations are used for pre- and postoperative evaluation but are not very predictable of the success of bone formation at an early stage. Bone formation relative to THA using PET bone scans has been studied [1, 38]. An imaging protocol [21] has been designed to evaluate the bone formation progress of patients who had a TSF affixed to the tibia.

The primary objective of this study was to determine whether videos of spatiotemporal uptake of ^18^F^−^ with PET bone scans could show problematic bone healing in patients with complex tibia conditions. A secondary objective was to determine if semiquantification of uptake, viewed as a quantity per day to make it comparable across patients, was consistent with bone healing.

## Patients and Methods

Between October 2013 and April 2015, 24 patients (six females, 18 males) with complex fractures, osteomyelitis, and osteotomies who had a TSF applied to the tibia agreed to participate in this approved (Regional Ethics Committee Number, 2012/1049 31/1) study and signed an informed consent statement. The mean age of the patients was 45 years (range, 17–78 years) (Table 1). Twenty-two patients were examined at a mean of 55 days (range, 39–103 days) after surgery and again at 111days (range, 81–184 days). Patient 21 died between the first and second scans and Patient 2 was examined only once to determine the amount and spatial distribution of bone formation shortly before TSF removal (Table 1). Patients 8 and 9 underwent revision surgery after their first two scans and were examined twice more after revision surgery. Patient 15 was treated with ultrasound after his second scan and underwent a third scan. Patient 5 had both tibiae corrected simultaneously and Patient 12 had both tibiae corrected serially, thus there were 26 legs examined (Table 1). The cohort consisted of eight patients with pseudarthrosis, seven with complex fractures, three with arthrodesis, and six with only osteotomies. However, some patients had two osteotomies and others had an osteotomy in conjunction with another condition totaling 16 osteotomies. Ten patients also had infections (Appendix 1. Supplemental material is available with the online version of *CORR* ^®^.).

**Table 1:** Patient descriptions

Patient number	Age (years)	Gender	Days to first PET/CT	Days to second PET/CT	Reason	Resolution	Days to resolution
1	64	M	274	N/A	Refractures in segmental left tibia	TSF extraction	328
1	64	M	43	148	New TSF as fractures not healing	TSF extraction healed	169
1	64	M	374	400	New fracture between former two fractures with no TSF attached	Former two fractures healing	N/A
2	36	M	135	N/A	Pseudarthrosis right lower leg	TSF extraction healed	211
3	52	M	40	84	Fracture healing in left leg	TSF extraction healed	167
4	44	M	50	122	Pseudarthrosis plus infection in right lower leg	TSF extraction healed	161
5	35	M	43	85	Genu varum–pseudoachondro-plasia. Both legs treated at the same time.	TSF extraction healed	182
6	17	F	52	94	Reduction malformation right leg	TSF extraction healed	345
7	31	M	48	129	Osteomyelitis, right lower leg fracture	Leg amputated, continued infection	226
8	28	M	60	184	Pseudarthrosis of left lower leg	Patient did not achieve healing, new operation	N/A
8	28	M	291	368	Reoperation, no new TSF was applied	TSF extraction healed, has resumed dancing lessons	417
9	45	F	50	91	Nonunion, pseudarthrosis of distal tibia, pilon fracture of right distal tibia	CT, nonunion; plane film radiograph not seen. Low 50-day uptake should have prompted revision	N/A
9	45	F	197	269	Reoperation, no new TSF was Applied	TSF extraction healed	329
10	33	M	32	92	Fracture, varus deformity & lengthening	TSF extraction healed	108
11	68	F	103	146	Wound autologous bone grafting & infection, arthrodesis	TSF extraction healed	216
12	35	M	49	104	Severe bowing deformities of the tibia, right leg	TSF extraction healed	152
12	36	M	43	84	Severe bowing deformities of the tibia, left leg	TSF extraction healed	184
13	30	M	42	88	Varus deformity & lengthening	TSF extraction healed	99
14	21	F	46	93	Genu valgum, valgus deformity	TSF extraction healed	114
15	52	M	53	95	Pseudarthrosis, osteotomy	Patient not achieving remodeling as expected	N/A
15	52	M	151	N/A	Ongoing TSF with ultrasound stimulation of bone	TSF extraction, wearing cast	518
16	40	M	148	188	Proximal tibia fracture, varus deformity, original scan delayed	TSF extraction healed, returned for a second scan 35 days after removal	152
17	70	M	50	84	Comminuted distal tibia fracture	TSF extraction healed	147
18	29	M	46	84	Comminuted distal tibia fracture	TSF extraction healed	199
19	64	M	40	81	Proximal osteotomy, distal pseudarthrosis (infected)	TSF extraction healed	386
20	58	M	42	83	Infected lower leg arthrodesis	TSF extraction healed	173
21	45	M	40	N/A	Fracture & infection	Died	57
22	78	F	39	70	Distal tibia & fibula fracture accompanied by diabetes mellitus	TSF extraction healed	128
23	23	F	53	90	Pseudarthrosis	TSF extraction healed	109
24	55	M	41	83	Pseudarthrosis	TSF extraction healed	153

Days are calculated from the surgery to attach the frame; PET = positron emission tomography; TSF = TAYLOR SPATIAL FRAME^TM^; M = male; F = female; N/A = not applicable.

### PET/CT Bone Scan

Because a PET scan provides a map of a particular physiologic function associated with a particular organ or tissue, it does not provide many anatomic clues regarding where the radionuclide uptake is located. Therefore, PET scans generally are accompanied by an anatomic scan such as CT. Hybrid PET/CT scanners create both scans in the same instrument serially, without the necessity of moving the patient. A clinical PET/CT scanner (Biograph^TM^ 64 TruePoint^TM^ TrueV; Siemens Medical Solutions, Erlangen, Germany) was used for all the examinations. The patients were given 70 mL of water to drink (to hydrate the body) before being placed supine on a scanning couch with both legs in the view. For each examination two MBq per kg body weight of ^18^F^−^ was injected. The imaging protocol used was described by Hatherly et al. [21]. To study the influx of radioactive material into the healing bone with time, a 45-minute dynamic acquisition was performed. This can be reconstructed as a time series of volumes, each representing a different time since injection of the ^18^F^−^. Forty-five minutes was chosen because this was a reasonable amount of time for the patient to lie still. Then, to capture the maximum uptake in bone, a 5-minute static scan was performed after 60 minutes [25, 26, 36]. A series of volumes was reconstructed for each patient, comprising five volumes at 1-minute intervals (thus the first 5 minutes postinjection), five at 3-minute intervals, three at 5-minute intervals, and one at 10 minutes later, totaling 45 minutes. Then, to better examine the early phase of radioactive uptake, a second series of volumes was reconstructed, comprising six volumes at 10-second intervals (thus the first one minute postinjection), four at 30-second intervals, seven at 1-minute intervals, five at 3-minute intervals, and four at 5-minute intervals, totaling 45 minutes. This was done for one dynamic scan sequence from each of four patients judged as representative of patients from the four categories of problems in the cohort (Patients 8, 11, 12, and 18).

Each PET voxel was 4.07 × 4.07 × 3.00 mm in size and each volume had an x-y matrix size of 168 × 168 with 74 slices in each volume. Because the patient was not moved during the entire acquisition, one noncontrast low-dose CT scan was used for attenuation correction of all reconstructions. However, in four instances, the patient had moved between the dynamic and static scans; therefore, a second CT scan was performed using a procedure identical to the first.

### Image Analysis

For each patient with multiple scans separated by time, the attenuation correction CT volume data from subsequent examinations were spatially aligned to bring the ends of the bone that were expected to heal (bone fragments) into alignment. This was done using a three-dimensional (3-D) custom-developed image processing tool which creates a 3-D coordinate transformation, described and validated elsewhere [15, 32, 33]. Once alignment using the CT volumes was done, the same transformation was used to align the corresponding PET volumes. As the alignment between the temporally separated CT volumes was not perfect and the registration between the PET and CT provided by the hybrid camera was not perfect, manual transformation (affine) techniques were used to refine the alignment between the PET scans [24]. This was necessary for 12 patients.

Each series of PET volumes was displayed using a color scale normalized to the highest voxel value for the series. The volumes were viewed as a time-sequenced video to observe the radioactivity entering the tibia and where it was taken up (or not) by the bone.

The radionuclide uptake of the healing bone also was semiquantified using standardized uptake values (SUVs). The SUV is calculated as the volume-derived concentration of ^18^F^−^ divided by the whole-body concentration of the injected activity per kilogram of body weight. Details of this calculation were reported by Thie [40]. To derive the SUV from the volume data, it is customary to place a volume of interest (VOI) around the 3-D region to be evaluated. In this case a 25-mm radius spheric VOI was chosen because it encompassed the bone fragments on all the patients. The SUV mean (SUVmean) (the mean value of all approximately 1317 voxels in the VOI) and SUV maximum (SUVmax) (the highest value of a voxel in the VOI) were calculated. To make the SUV data comparable over all the patients, the difference per day (SUVmaxDPD and SUVmeanDPD) between successive PET/CT scans were calculated. In some instances, the patient started to heal more rapidly between the first and second scans than between the operation and the first scan, thus producing a negative SUVmaxDPD and SUVmeanDPD. This SUV difference data were used in an effort to determine if it could be related to the duration of the bone healing. All PET/CT volumes, used in the SUV calculations, were aligned with a maximum spatial error within one CT voxel (3.6 mm), thus assuring that the same VOI region was used for the SUV calculation despite the PET scans being done on different dates. The SUVmax and SUVmean semiquantitative data for all patients for each complex problem and SUVmaxDPD and SUVmeanDPD were calculated. The SUVmax data also were calculated for a normal bone segment not involved in the bone healing, reflecting normal bone turnover as promoted by the normal balance of osteoclastic and osteoblastic activity. In this way the patient’s own normal bone turnover is used as a control for the turnover in the healing bone (Table 2).

**Table 2:** Summary of findings for all patients

Patient number	Days first PET/CT	SUV		Nonoperated leg SUVmax	Days second PET/CT	SUV		Nonoperated leg SUVmax	SUV difference per day		Days to TSF removal
		Maximum	Mean			Maximum	Mean		Maximum	Mean	
1 Upper	274	35.16	7.70	1.82	N/A	N/A	N/A	N/A	N/A	N/A	328
1-Lower	274	18.95	5.50	1.82	N/A	N/A	N/A	N/A	N/A	N/A	328
1-Lower	43	35.05	7.98	2.40	148	39.67	10.09	2.10	−0.044	−0.020	169
2	135	23.59	6.94	1.34	N/A	N/A	N/A	N/A	N/A	N/A	211
3	40	60.77	19.80	2.21	84	47.23	15.86	2.46	0.308	0.089	167
4	50	49.80	17.97	2.88	122	34.51	13.02	1.92	0.212	0.069	161
5-R	43	21.86	4.65	2.12	85	40.71	5.42	4.86	−0.449	−0.018	182
5-L	43	25.91	5.08	2.98	85	30.88	7.08	4.31	−0.118	−0.048	182
6	52	40.62	9.29	3.60	94	33.54	7.36	2.92	0.169	0.046	345
7	48	25.46	8.85	2.19	129	22.11	9.27	2.36	0.041	−0.005	N/A
8	60	27.27	5.67	1.73	184	29.60	6.52	1.26	−0.019	−0.007	N/A
8	291	32.73	8.57	1.95	368	20.80	4.04	1.07	0.155	0.059	417
9	50	17.46	2.85	1.43	91	18.13	3.63	2.13	−0.016	−0.019	N/A
9	197	18.71	3.53	2.91	269	22.81	3.76	2.80	−0.057	−0.003	329
10	43	68.55	20.02	1.86	92	63.27	23.60	1.04	0.108	−0.073	108
11	103	49.13	11.54	1.92	146	25.13	6.72	1.18	0.558	0.112	216
12-R	49	43.50	10.68	2.11	104	27.15	11.39	2.30	0.297	−0.013	152
12-L	43	26.83	7.19	3.85	84	33.17	8.45	3.22	−0.155	−0.031	184
13-Upper	42	20.51	5.36	1.40	88	17.93	5.11	2.30	0.056	0.005	99
13-Lower	42	17.58	3.61	1.38	88	32.61	6.46	1.89	−0.327	−0.062	99
14-Upper	46	70.26	13.23	1.40	93	13.85	5.22	2.30	1.200	0.171	114
14-Lower	46	47.18	9.11	1.38	93	29.98	5.25	1.89	0.366	0.082	114
15-Upper	53	26.37	7.95	1.43	95	28.13	6.33	2.20	−0.042	0.039	N/A
15-Upper	151	19.18	3.60	2.69	N/A	N/A	N/A	N/A	0.073	0.044	518
15-Lower	53	31.91	10.26	1.43	95	34.31	12.74	2.20	−0.057	−0.059	N/A
15-Lower	151	21.16	7.78	0.07	N/A	N/A	N/A	N/A	0.110	0.025	518
16	148	31.63	10.18	2.92	188	24.78	9.03	2.85	0.171	0.022	152
17	50	29.62	10.00	2.97	84	25.79	7.56	1.90	0.113	0.072	147
18	46	14.29	3.59	1.01	84	12.78	3.98	1.09	0.040	−0.010	199
19-Upper	40	19.90	5.25	1.98	81	13.29	4.00	1.49	0.161	0.030	386
19-Lower	40	26.53	11.53	1.98	81	19.49	6.64	1.49	0.172	0.119	386
20	42	39.04	14.04	1.36	83	36.06	12.16	1.34	−0.073	−0.046	173
21	40	46.80	21.74	1.59	N/A	N/A	N/A	N/A	N/A	N/A	N/A
22	50	26.90	9.56	1.59	81	62.33	15.09	1.19	−1.43	−0.178	139
23	42	27.86	8.48	2.98	79	20.68	6.75	2.60	0.194	0.047	98
24	29	25.31	13.77	1.07	71	17.04	7.85	2.16	0.197	0.141	156

PET = positron emission tomography; SUV = standardized uptake value; SUVmax = standardized uptake value maximum; TSF = TAYLOR SPATIAL FRAME^TM^; Upper = proximal tibia; Lower = distal tibia; L = left leg; R = right leg; all SUV values calculated from the 5-minute scan after 60 minutes; all days are calculated from the operation to attach the TSF; N/A = not applicable.

Using the first 5-minute static scan of each patient (viewed with the CT and PET volumes superimposed), the VOI was placed around the bone fragments and then was superimposed on the sequence-matched volume. The VOI typically includes some surrounding tissue and muscle. To compare the SUVmax for the tibia being treated with those in the nontreated bone, the same-sized VOI was placed over normal nonbroken bone in the same location in the contralateral leg, which, for 22 patients, was also in the PET/CT scan. However, for the two patients who had both tibiae treated, a nontreated part of each tibia exhibiting radionuclide uptake comparable to that in untreated bone was chosen.

Because we had a heterogeneous patient population, some of whom had infections, principal component analysis (PCA) was used to determine the major factors affecting healing. These are shown as vectors which represent the factors such as condition (pseudarthosis, fracture, arthrodesis, and osteotomy), infection, amount of uptake in the healing bone (evaluated by the SUV difference per day), and the time between TSF application and removal. The calculations were performed using R version 3.1 (R Foundation for Statistical Computing, Vienna, Austria) [34].

## Results

**Fig. 1A–F:**
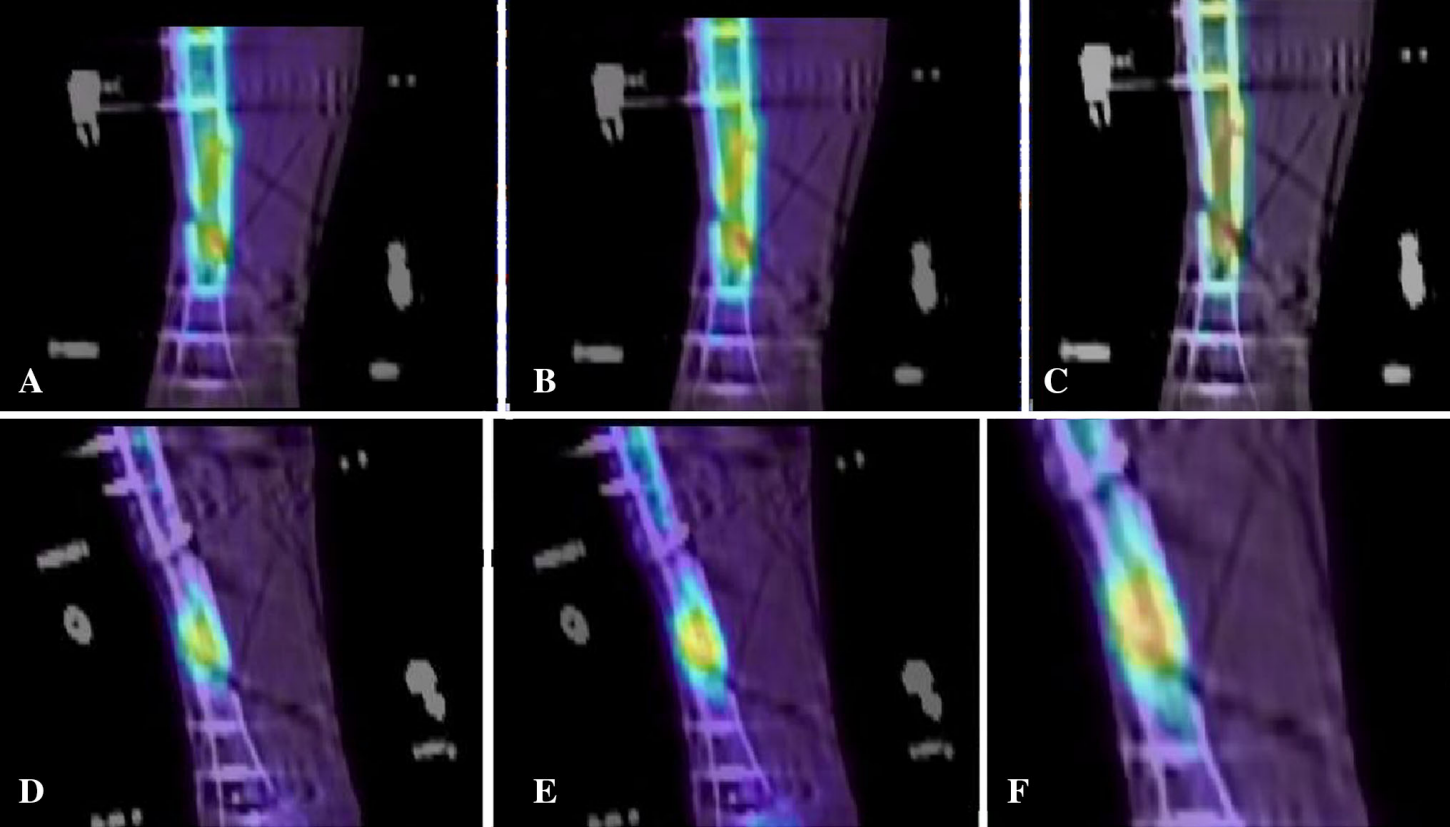
^18^F^−^ uptake is pictured for Patient 8. At the first 6-week scan before revision surgery, the uptake at (**A**) 20 minutes after injection, (**B**) 30 minutes after injection, and (**C**) 45 minutes after injection, appears uneven. At the 6-week scan after revision surgery, at (**D**) 20 minutes after injection, (**E**) 30 minutes after injection, and (**F**) 45 minutes after injection, bone formation appears to be more uniform.

**Fig. 2A–D:**
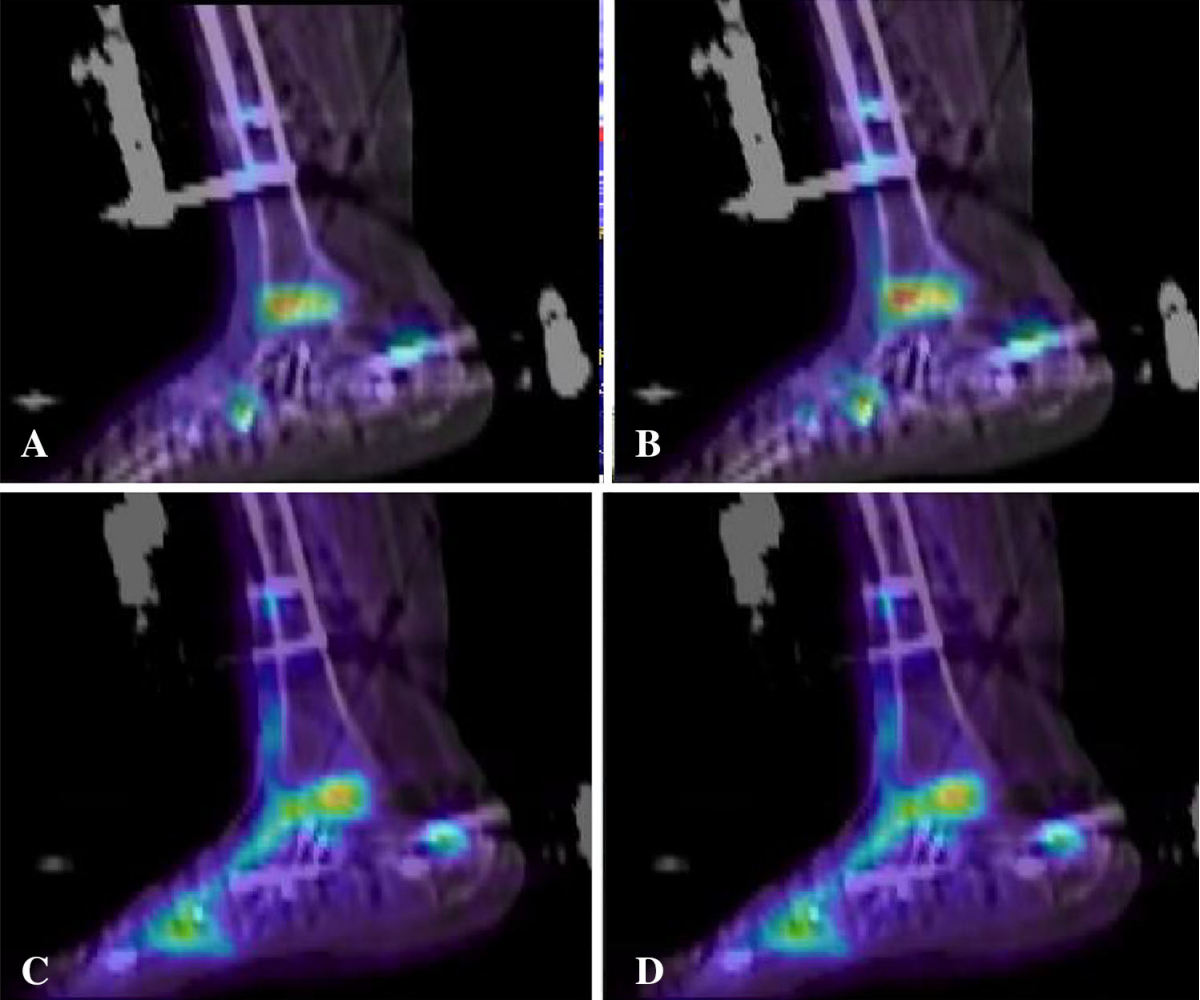
^18^F^−^ uptake is pictured for Patient 11. At the first 6-week scan, the uptake, at (**A**) 20 minutes after injection, and (**B**) 30 minutes after injection appears to be evenly distributed across the arthrodesis. At the 6-week scan after revision, at (**C**) 20 minutes after injection, and (**D**) 30 minutes after injection, bone formation appears to still be uniformly distributed.

**Fig. 3A–D:**
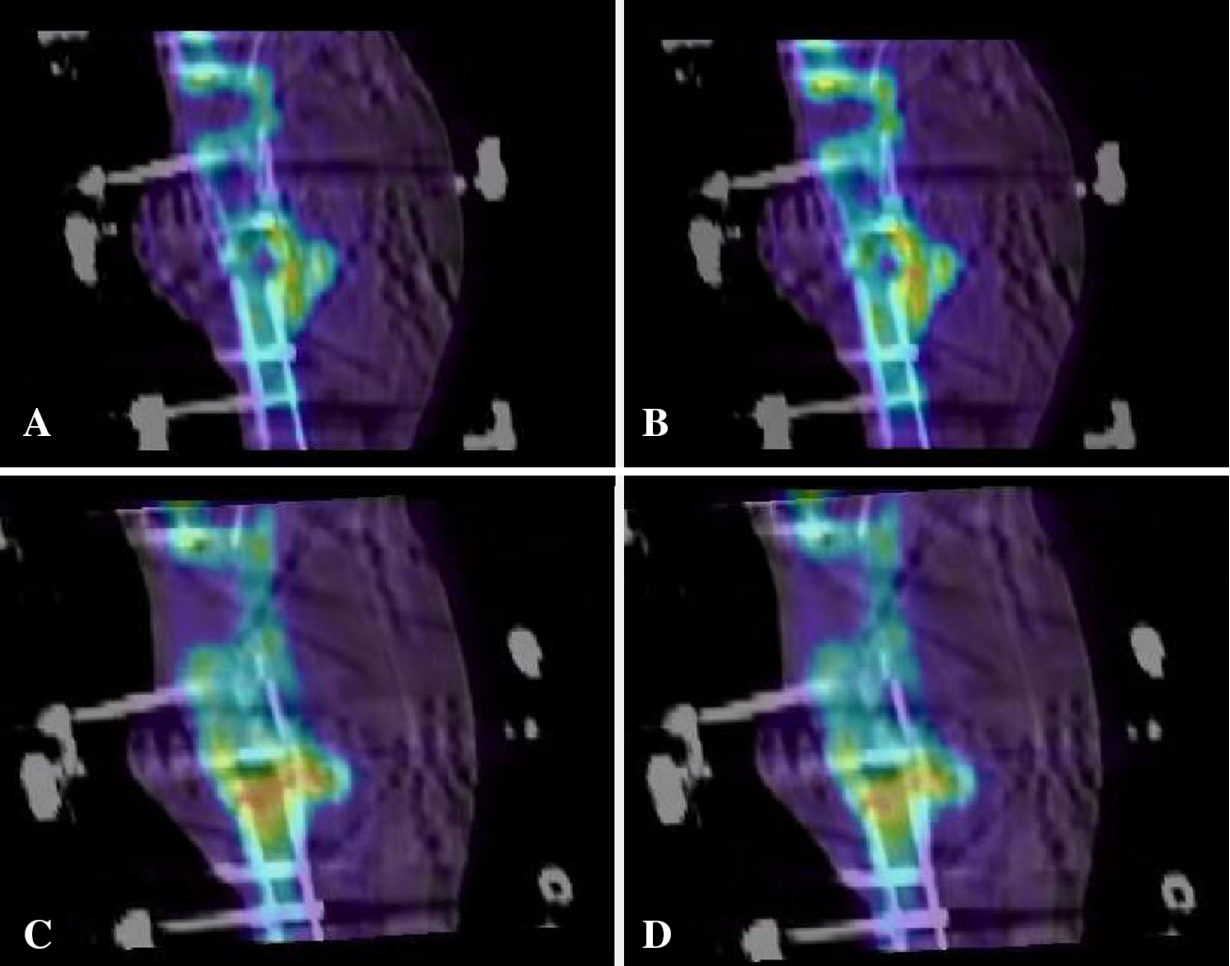
^18^F^−^ uptake is pictured for Patient 15. At the first 6-week scan before ultrasound stimulation, the uptake at (**A**) 20 minutes after injection and (**B**) 30 minutes after injection, appears uneven especially across the distal pseudarthrosis. At the 6-week scan after ultrasound stimulation, (**C**) 20 minutes after injection and (**D**) 30 minutes after injection, bone formation across the distal pseudarthrosis and the proximal osteotomy appears to be more uniform.

**Fig. 4A–D:**
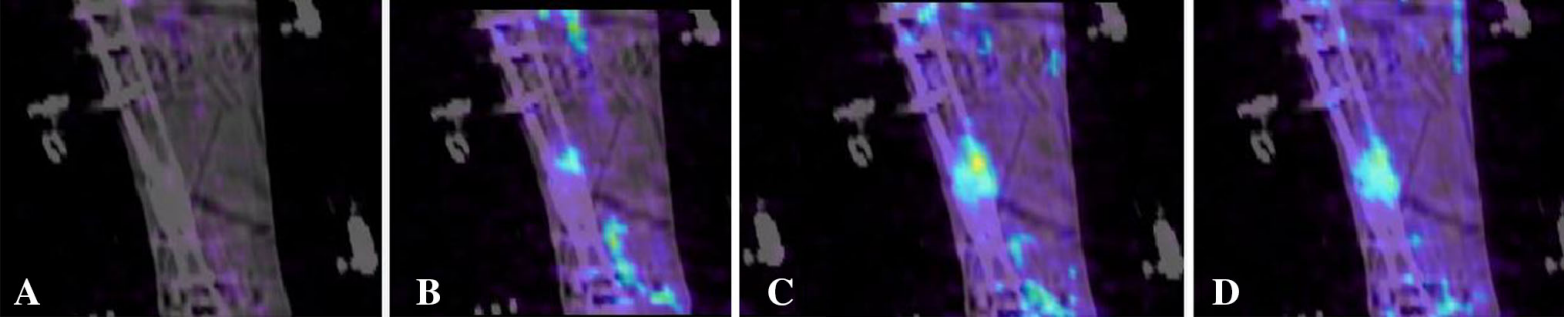
Six weeks after revision surgery, ^18^F - uptake for Patient 8 at (**A**) 30 seconds, (**B**) 40 seconds, (**C**) 50 seconds, and (**D**) 60 seconds after injection of the radionuclide is shown. The illustrations show that there was rapid uptake of radioactive material at the location of the pseudarthrosis and good blood flow, because after 30 seconds some uptake was present, but at 60 seconds a noticeable amount of radioactive uptake was present.

The main objective of this study was to identify the spatiotemporal distribution of ^18^F^−^ uptake in healing bone at different times during the healing process by observing the uptake as a video. In this publication, static images derived from the videos are used to illustrate normal and abnormal uptake. For example, for Patient 8, the first PET scan (Fig. 1A at 20 minutes after injection, Fig. 1B at 30 minutes, and Fig. 1C at 45 minutes) (Video 1. Supplemental materials are available with the online version of *CORR* ^®^.) shows that the radioactivity is unevenly distributed across the healing bone, indicating that the bone formation is not progressing as desired, thus identifying a need for revision. After revision surgery, in the third scan it can be seen that the radioactivity is evenly distributed (Fig. 1D again at 20 minutes after injection, Fig. 1E at 30 minutes, and Fig. 1F at 45 minutes) (Video 2. Supplemental materials are available with the online version of *CORR* ^®^.). Good healing across the arthrodesis is illustrated in Patient 11’s first (Fig. 2A at 20 minutes after injection and Fig. 2B at 30 minutes) (Video 3. Supplemental materials are available with the online version of *CORR* ^®^.) and second PET scans (Fig. 2C again at 20 minutes after injection and Fig. 2D at 30 minutes) (Video 4. Supplemental materials are available with the online version of *CORR* ^®^.). Improvement in bone formation evenness can be appreciated between the first (Fig. 3A at 20 minutes after injection and Fig. 3B at 30 minutes) (Video 5. Supplemental materials are available with the online version of *CORR* ^®^.) and third (Fig. 3C again at 20 minutes after injection and Fig. 3D at 30 minutes) (Video 6. Supplemental materials are available with the online version of *CORR* ^®^.) scans of Patient 15. This particular patient had ultrasound stimulation between the second and third scans. To show the rapid uptake of ^18^F^−^, Patient 8’s third scan is shown at 30, 40, 50, and 60 seconds after injection (Fig. 4) (Video 7. Supplemental materials are available with the online version of *CORR* ^®^.). These images show that a considerable amount of radioactivity has already been taken up in the healing bone even after as short a time as 1 minute, indicating the rapid uptake by bone of ^18^F^−^ when undergoing bone formation. The early uptake (Fig. 4) also shows that, in this case, there is good blood flow to the bone, which is important to promote healing.

**Fig. 5:**
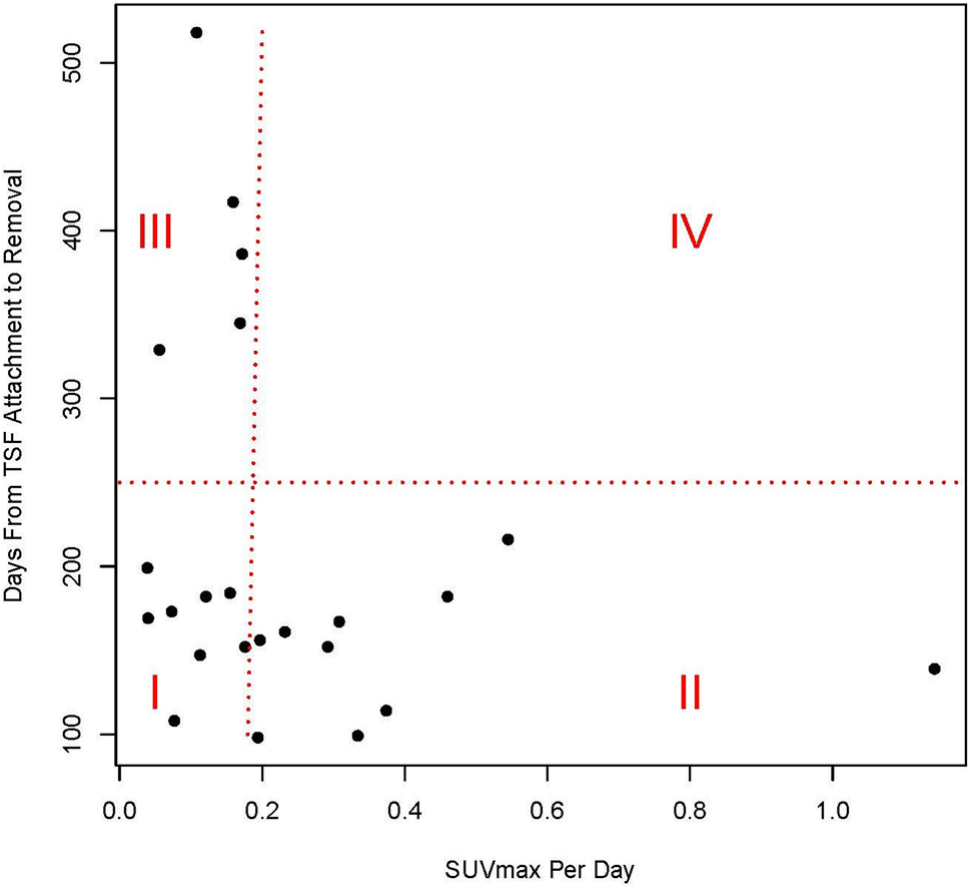
This plot shows the standardized uptake value maximum difference per day (SUVmaxDPD) versus the days until TSF removal. It is divided into four regions which illustrate the data shown in Table 2. Region I represents patients with an SUVmaxDPD less than 0.18, but who achieved healing in less than 250 days. Region II represents patients with an SUVmaxDPD of 0.18 or greater and who achieved healing in less than 250 days. Region III represents patients whose SUVmaxDPD was less than 0.18 and who needed more than 250 days to achieve healing. No patients were in Region IV where the SUVmaxDPD was greater than 0.18 and the patient took more than 250 days to achieve healing.

**Fig. 6:**
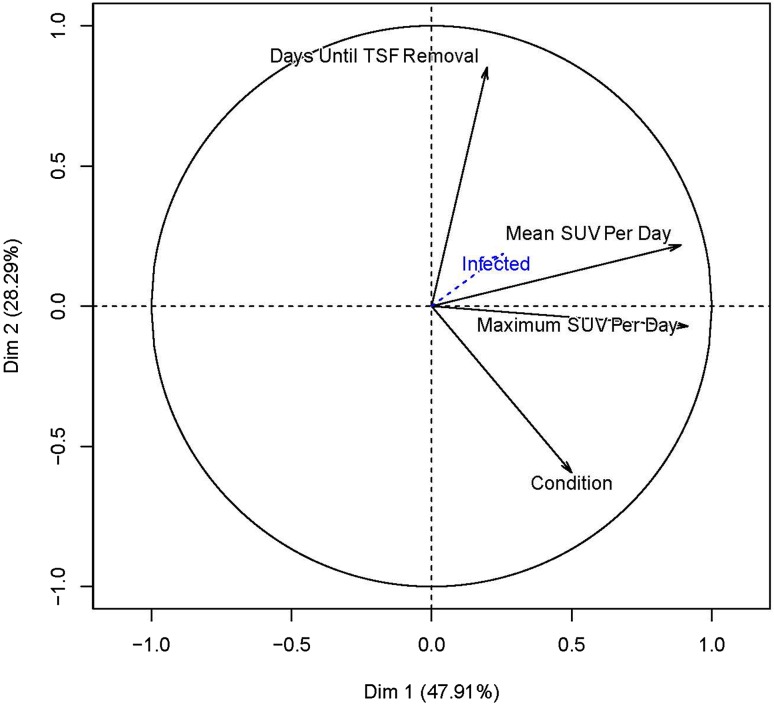
A factor map derived from principal component analysis is shown. The factors representing the standardized uptake value maximum difference per day (SUVmaxDPD) and standardized uptake value mean difference per day (SUVmeanDPD) are the most important factors relative to the days until TSF removal as these are the longest vectors and each is approximately perpendicular to the days until TSF removal vector. The particular problem that the patient had (Condition) was a strong factor. The vector representing infection is very small, showing that in this patient population, infection was not a strong factor affecting healing.

**Fig. 7:**
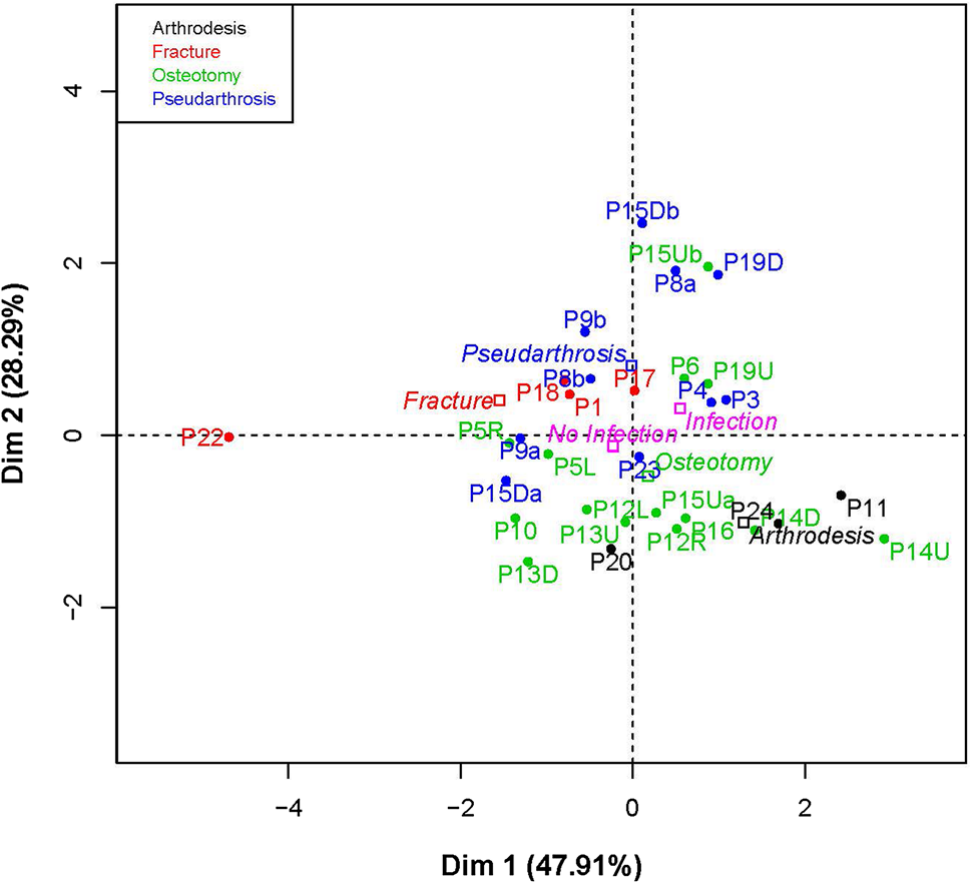
This plot pairs the patient and their respective condition in relation to the standardized uptake value maximum difference per day (SUVmaxDPD) value and the days to TSF removal (recovery). The plot is color coded with respect to the conditions represented in this patient population. The patients are designated by P and the patient number, ie, P15 = Patient 15; R = right; L = left; U = proximal tibia; distal tibia = D. Where there are more than one scan pair (Patients 8, 9, and 15; Table 2), the different pairings are denoted as a, and b.

The secondary purpose of this study was to investigate if the absolute magnitude of the SUVmaxDPD and the SUVmeanDPD could be used to predict that bone healing is progressing in a satisfactory manner. Looking at the data, it was found that the absolute magnitude of the SUVmaxDPD and SUVmeanDPD data exhibited a relationship with the number of days between TSF attachment and removal (Table 2; Fig. 5). As can be seen in Fig. 5, there appears to be a clear delineation at a SUVmaxDPD value of 0.18. Of the 16 patients who progressed rapidly toward healing (less than 250 days before removal of the TSF), in 10 instances the absolute magnitude of the SUVmaxDPD was equal to 0.18 or greater, but in eight instances it was less than 0.18. For the remaining five patients the bone healed in more than 250 days (Table 2; Fig. 5). There are 23 points on the graph, as Patient 12 had both legs corrected serially and Patient 5 had both legs corrected simultaneously (one leg each in Region I and Region II making two extra points on the graph). Patients 2 and 21 had only one scan, and Patient 7 did not complete treatment so these patients are not included on the graph. Additionally, three of the patients who were slow to achieve healing underwent revision surgery. For Patient 5 who had both tibiae treated simultaneously, the right tibia progressed more rapidly toward healing (SUVmaxDPD was −0.46 for the right leg, −0.12 for the left), consistent with clinical observation (Appendix 1. Supplemental materials are available with the online version of *CORR* ^®^.). The PCA factor plot (Fig. 6) shows that the SUVmeanDPD or SUVmaxDPD values comprise the largest factors in predicting good bone healing. The two factors (SUVmaxDPD and SUVmeanDPD) are the most important factors relative to the Days to TSF Removal factor as they are the longest vectors and each SUV factor is approximately perpendicular to Days to TSF Removal factor. The infection vector is very small, meaning that it does not influence the outcome very much. For all except Patient 7, infection was successfully resolved. When the different patient conditions were plotted individually (not shown), the osteotomies were the strongest factor, followed by pseudarthrosis which makes sense as these were most represented in this patient cohort. Also shown is an individual PCA factor map for each patient’s condition(s) (Fig. 7). This plot pairs the patient and condition in relation to the SUVmaxDPD and the days to TSF removal. The plot is color coded with respect to the patients’ conditions shown in Table 2.

## Discussion

A key problem when treating patients with complex fractures with a TSF was knowing whether the treatment was effective, preferably as early as possible. A second key problem was knowing when the TSF can be removed, as removal too early leads to complications, while leaving the frame on longer than necessary negatively affects the patient’s life and unnecessarily increases the risk of infections. It was shown in previous studies [11, 13, 16] that ^18^F^−^ PET bone scans can reflect the physiologic turnover of bone. In the current study, videos derived from ^18^F^−^ dynamic scans were used to provide a spatiotemporal map of the activity at the ends of the bone that were supposed to heal. This activity or lack thereof is proportional to bone formation, thus directly indicating where and how the bone is healing. The spatiotemporal data provided by the videos were found to be very informative, especially for three patients in whom the bone fragments were not healing as expected. When this occurs at the time of the first PET scan, this potentially can prompt remediation. Additionally, semiquantitative SUV difference data were examined to see if these were related to the days between TSF application and removal. We found that an absolute value of 0.18 or greater for the SUVmaxDPD corresponded in this study to rapid bone healing (Region II, Fig. 5). No patients in this cohort with a high absolute value of SUVmaxDPD took a long time to achieve healing (Region IV, Fig. 5). However, some of the patients with a lower absolute value of SUVmaxDPD took a long time to achieve healing (Region III, Fig. 5), while some achieved healing in a shorter time (Region I, Fig. 5), which implies that one cannot simply look at the value of SUVmaxDPD, but rather also must examine spatiotemporal uptake. Moreover, this study showed the potential advantages of dynamic and static PET acquisition, because this allowed the semiquantitative analysis used in an earlier study [25] and the reconstruction of short-duration volumes to observe the dynamics of ^18^F^−^ uptake as was done in the current study.

The limitations of this study were the small number of patients and the heterogeneous cohort (regarding the reason for the TSF treatment). Although there were 16 osteotomies, some were done for lengthening or deformity correction only and some were done for both. In this cohort it did not seem that infection was a major factor in the patients’ progress (Fig. 6). However, as described in a previous study [24], the effect of infection on the radionuclide uptake is to increase the uptake rate early during the process followed by a decrease in uptake rate, in contrast to a noninfected bone. Another limitation of this study was that only two PET scans were included in the protocol. Additional PET scans at 18 weeks and at 6 months might provide more information, especially regarding predicting when the frame could be removed. It is true that one dynamic scan acquired shortly after the operation, from which one video could be produced, should give the surgeon an indication of good healing or not, but as this technique is quite new, a larger cohort needs to be examined. To use the semiquantitative data in the form of the SUV, at least two PET/CT scans are necessary. Use of the SUV as a definitive value is much more problematic (Fig. 5). Finally, although PET/CT scans are more costly than a CT scan alone, this procedure can substitute for one of the routine CT or radiographic procedures. If it can be shown that a PET/CT scan is valuable to the patient, especially in terms of early intervention if the healing does not appear to be progressing, or confirming that the healing has progressed to the point where the TSF can be removed without the possibility of the bone breaking again, then the benefits could outweigh the cost.

Dynamic observation together with measuring SUVmax before and after different adjuvant treatments may be used to evaluate the quality of bone formation in a particular patient. In this study it was shown that during the course of treatment, a persistent low uptake as measured by SUVmax, and/or uneven uptake observed using a time-sequenced video shortly after surgery, could indicate poor healing potential. This could prompt early intervention either surgically, with bone grafting, to add stability, and/or compression/distraction of the regenerate as was done for Patients 8 and 9. With respect to Patients 1, 8, and 9, our experience with the method was early and we did not know if the data could be trusted, so the TSF was removed for Patient 1 and revision was delayed for Patients 8 and 9. For Patient 15, who first requested an amputation, ultrasound stimulation was used. Encouraged by the success of the treatment (Fig. 3), the patient achieved complete healing. However, although successful in this particular patient, Griffin et al. [17] reported that these treatments are not always successful.

In our longitudinal study, only diagnostic, as opposed to therapeutic, quantities of fluoride were used with the ^18^F^−^ ion reflecting only bone formation. Cheng et al. [9], in their evaluation of bone formation with ^18^F^−^ fluoride, state “Radionuclide tracers such as ^18^F^−^ fluoride bind to newly mineralizing bone, thus serving as a marker of bone blood flow and for osteoblastic activity”, and they cite Reeve et al. as providing this information [35]. There is no indication that ^18^F^−^ in the concentrations used in these procedures (approximately 8 × 10^−9^ mg/L in the blood and for an SUV of 20 corresponding to 20 × 10^−3^ mg/L in the region with maximum uptake in the VOI of approximately 65 mL) has any effect on osteoclastic activity (Everett [12] reported enhanced osteoclast function at 1 mg/L but was unable to determine if this was “a direct effect or indirect via an action on residual osteoblasts present in the culture system”). From these ^18^F^−^ bone scans we are unable to evaluate bone resorption, that is, osteoclastic activity, as ^18^F^−^ is incorporated into the apatite crystal [18] . If it were desired to study resorption, then potentially one could do a differential study before and after using gallium to suppress osteoclastic bone resorption [41].

Because of the high uptake resulting from bone formation, the injected activity was approximately half that used when studying a normal skeleton. For example, Win and Aparici [42] reported injecting 5.4 to 4.1 MBq per kg for patients weighing 65 to 108 kg when performing normal bone studies. They also described positive correlations between SUV in the lumbar spine and height and weight but no significant correlation between SUV and serum creatinine or age (although they pointed out later that this may be the result of the mean age of their patients). It also was shown in a study using rats that the effect of ^18^F^−^ in bone is not influenced by genetics [31].

Because this technique directly reveals bone formation, it can be used in any adult patient when there is a question regarding whether bone formation is progressing as expected. This technique is not limited to patients with the TSF, but is applicable to a wide variety of orthopaedic patients. Bastawrous et al. [4] pointed to the use of ^18^F^−^ NaF for predicting bone viability and bone healing after surgery or trauma, diagnosis of fracture nonunion or delayed union, and quantitative assessment of bone metabolism. Moreover, at any hospital that does PET/CT studies these patients can be studied at the beginning of the day before other patients are scanned, as unlike studies with ^18^F^−^ fluorodeoxyglucose, there is no need to wait for 1 hour after injection of the radionuclide before scanning.

Our study leads to numerous clinical questions that it would be useful to answer. One question is: Could ^18^F^−^ bone scans be used to evaluate if the bone has healed sufficiently that the TSF can be removed without a high probability of the bone breaking again, as occurred in Patient 1? If so, then ^18^F^−^ bone scans could be a valuable aid to the surgeon in their decision to remove the TSF. Another question is: Could ^18^F^−^ bone scanning be used in evaluating whether application of (varying) pressure through the TSF enhances bone healing? Another question is: Could a sensitive detector, which can measure radioactivity from a naturally occurring tracer which is taken up by reforming bone such as 90Sr, be used instead of imaging to aid in the clinical decision to remove the TSF [30]?

As future work we would like to restudy patients with a clinical ^18^F^−^ bone scan at the time of removal of the TSF to ensure that the bone is sufficiently healed (as was done for Patients 1 and 2). It also might be useful to perform an additional ^18^F^−^ bone scan approximately 6 months after a patient’s treatment has ended to see if above-normal bone formation is still occurring [20]. Although not performed at exactly 6 months, this was done for Patients 1 (210 and 236 days in conjunction with another fracture between the original two) and 12 (370 and 411 days in conjunction with the treatment of the left leg). For these patients, the SUV of the VOI over the healing bone was still above that of normal bone, indicating that the bone formation was occurring at a greater rate than expected for normal bone. A future study is needed to determine if the injected activity can be reduced without reducing the clinical information that is produced, hence enabling more scans during the course of the treatment. Some preliminary work (done by randomly subsampling the dynamic data) suggests that at least a 50% reduction in injected activity (thus reducing the effective dose to the patient) is possible without losing any image quality or compromising the numeric results [24].

Unlike planar radiography/CT morphologic data, the use of PET in this study to observe the spatiotemporal uptake by the bone fragments, although limited to a small number of patients, has allowed us to study more directly how and where bone formation occurs from a functional point of view. An important point shown in this study and in previous studies [25, 26] is that ^18^F^−^ bone scans can provide semiquantitative and qualitative functional information regarding bone formation in each part of the bone [5, 14, 37]. The current study shows that, in clinical practice, there are numerous situations in which a functional (or biologic or metabolic) assessment of bone formation may be of value. Because the ^18^F^−^ bone scan gives information regarding bone turnover in each part of the regenerate, a preoperative ^18^F^−^ bone scan potentially could aid in preoperative planning not only to determine the viability of the bone, but also to evaluate the healing potential and need for additional stability or induction with, for example, an autologous bone graft. Each patient usually has a series of CT studies during the course of their treatment to assess their progress; the CT scan taken as part of the PET/CT protocol provides this same CT data. The advantage that PET provides over planar radiography or CT is that it adds spatiotemporal information regarding the biologic uptake of ^18^F^−^, however the study itself takes longer (45–65 minutes for the PET scan while planar radiography or CT studies take on average 15 minutes). We found this study to be valuable because it identified many potential clinical applications of ^18^F^−^ bone scans that could be valuable in following patients’ bone healing progress.

## Electronic supplementary material

Below is the link to the electronic supplementary material.

Supplementary material 1 (DOC 54 kb)

Supplementary material 2 (MP4 331 kb)

Supplementary material 3 (MP4 360 kb)

Supplementary material 4 (MP4 307 kb)

Supplementary material 5 (MP4 344 kb)

Supplementary material 6 (MP4 448 kb)

Supplementary material 7 (MP4 520 kb)

Supplementary material 8 (MP4 958 kb)
