# Supplementary material for: Can Spatiotemporal Fluoride (18F−) Uptake be Used to Assess Bone Formation in the Tibia? A Longitudinal Study Using PET/CT
Source: Clin Orthop Relat Res. 2017 Feb 1;475(5):1486–98. doi: 10.1007/s11999-017-5250-8 (PMC5384929; doi:10.1007/s11999-017-5250-8)
Supplement: Supplementary file 1 — Supplementary material 1 (DOC 54 kb) [file 11999_2017_5250_MOESM1_ESM.doc]

**Appendix 1**. Background information for the patients

**Patient 1** had refractures in his open segmental tibial fracture. He was treated with fixation of both fractures in a two-level TSF without revision. The first ^18^F^-^ study was performed to aid the decision to extract the frame. The PET/CT indicated ongoing high bone turnover indicating ongoing healing of the bone, confirmed by the morphologic distribution of uptake. The TSF was removed after 328 days at the patient’s request, a cast was applied, but subsequently the patient had a refracture in the intermediate fragment and a varus dislocation that required further treatment with a TSF. The refracture was “activated” by drilling and a proximal osteotomy was done for gradual correction of the varus deformity and slight lengthening. This second TSF was removed after 169 days. The patient again experienced a fracture in the intermediate segment that this time was treated with an intramedullary nail. For clinical reasons, he had two more PET/CT examinations at 374 and 400 days from attachment of the second TSF, allowing the study of the ongoing tibia remodeling after removal of the TSF. He is doing well.

**Patient 2** was referred to us with a nonunion of a tibial fracture. The plate and a 5 x 2 cm sequester (dead bone) was removed. He then had bone grafting and a TSF was applied. He was pain free wearing the frame but healing was very slow. Twelve months after the first operation a conventional radiograph showed healing of the fracture. At frame removal it was evident that the fracture was unstable. The fracture site therefore was opened and revised and the frame was not removed. He underwent frame removal at 211 days after the fracture site was revised and has been stable after that.

**Patient 3** had a malunion of an open tibia fracture. This originally was treated with a nail and healed in varus and 4 cm of shortening after two reoperations and bone grafting. He underwent correction and lengthening with a TSF with a distraction rate of 1 mm a day on the concave side. He showed poor bone regeneration and therefore underwent two reoperations with stabilization of the frame and bone grafting. The patient was included in the study before the last revision. Owing to previous failures, this patient was also treated with a Physio-Stim® (Orthofix, Lewisville, TX, USA), which is a device mimicking the electromagnetic field of a healing fracture. The fracture healed and the frame was removed 167 days after the index operation.

**Patient 4** was referred to us because he had an infected nonunion. A thorough revision, subsequent bone grafting, and application of a TSF were performed. The patient refused extraction of the fibular plate and acute shortening which probably prolonged the course of treatment. His infection was successfully eradicated but the healing was very slow. He was included in the study before his last revision surgery with bone grafting. The fracture healed, with frame removal 161 days after the index operation.

**Patient 5** had severely deformed and short limbs attributable to pseudoachondroplasia. He was included in the study when we performed bilateral proximal tibial osteotomies and application of a TSF for lengthening and correction of severe varus and rotation. The correction was finished earlier on the right leg owing to a residual deformity on the left side. Both frames were removed at 182 days.

**Patient 6** had a reduction malformation of the right leg. She had application of a TSF and a percutaneous osteotomy of the tibia with a Gigli saw and an oblique osteotomy of the fibula with an oscillating saw. One week after the operation lengthening was started with a rate of 1 mm a day. Owing to ankle contracture 5 weeks postoperatively she underwent a 5-day pause of the lengthening. Two months postoperatively we noticed that her fibula had healed prematurely and she therefore underwent reosteotomy. The distribution of the areas of maximum uptake was different in the tibial regenerate compared with in the fibula. In the tibia we observed three areas of high uptake whereas in the fibula there was only one area of maximum uptake. She achieved successful healing and the frame was removed 345 days after the initial operation.

**Patient 7** had a Gustilo IIIB open fracture to the distal tibia and underwent fixation first with a plate and screws. After three free flaps it was evident that he had a deep infection and therefore underwent a resection of dead bone, extraction of hardware, and fixation with a TSF. A preoperative PET/CT might have indicated that more bone should have been resected. The low uptake at 6 weeks should have prompted an early revision and bone grafting. Recurrent infections led this patient to decide in favor of an amputation.

**Patient 8** sustained a gunshot wound to the distal third of his left tibia and fibula. The fracture was fixed with an intramedullary nail and the patient presented 5 months later to the reconstruction section with an infected pseudarthrosis and a foreign body remaining in the soft tissue. He underwent intramedullary reaming, extraction of the foreign body, application of gentamicin, and fixation with a TSF. He was followed with plain film radiographs and was fully weightbearing and pain free. However, a CT scan showed a hypertrophic nonunion. Two hundred forty-four days after the original operation, he underwent an osteotomy for lengthening of the tibia proximally, bone grafting, and compression and stabilization of the nonunion, without removal of the original TSF. He had the TSF removed at 417 days and commenced dancing lessons.

**Patient 9** had a nonunion of the distal tibia after a pilon fracture that was fixed with plate and screws. She underwent hardware removal, revitalization (curettage) of the fracture site, and fixation with a TSF. Standard radiographs were not able to identify any healing disturbances. However a CT scan clearly showed a nonunion. The low uptake at 6 weeks maybe should have been a signal that an early revision with autologous bone graft would have been beneficial to the patient. One hundred fifty-four days after the original operation, she underwent bone grafting, stabilization, and fibular osteotomy without removal of the original TSF. She had the TSF removed at 329 days.

**Patient 10** was an otherwise healthy 33-year-old man who sustained a comminuted proximal tibial fracture after a gunshot wound in July 2009. He initially was treated with a temporary external fixator and a fasciotomy was performed. The wound was treated with vacuum-assisted closure and the fracture was fixed with a bridge plate and thereafter a secondary suture of the wound. Owing to delayed union the plate broke and was replaced in combination with an autologous bone graft. In October 2012 he presented with increasing medial pain, a 19^o^ varus deformity, and a 15-mm leg length discrepancy. In August 2013 he underwent a high tibial osteotomy that was fixed in a TSF circular frame, for gradual correction of the deformity. The patient was very distressed by the frame but achieved uneventful healing and the frame was removed after 108 days.

**Patient 11** is a 68-year-old woman with rheumatoid arthritis. After numerous operations of her left foot, she had an ankle endoprosthesis placed in her left ankle on September 2, 2013. Owing to postoperative infection the prosthesis had to be removed and the foot and ankle were held in a temporary external fixator (Hoffmann II^TM^; Stryker, Kalamazoo, MI, USA). The infection and wound were treated with antibiotics and a vacuum-assisted closure, respectively. On October 12, 2013, she underwent autologous bone grafting and fixation in a TSF circular frame. The frame was removed after 216 days. She is now fully weightbearing with minimal pain and the anterior wound has almost healed.

**Patient 12** is a 35-year-old man with hypophosphatemic rachitis. Owing to hydronephrosis, he has kidney failure and receives hemodialysis three times a week. He presented with severe bowing deformities of both tibias and femurs. At the time he was smoking 10 cigarettes a day. He previously had a femoral osteotomy of his left femur which was fixed with an intramedullary nail with a residual varus deformity. Surgery of his right femur and tibia was performed on December 12, 2013. The femur was fixed with an intramedullary nail whereas the tibia was fixed in a TSF for gradual correction. He achieved uneventful healing and after 152 days the frame was removed. His left leg tibia subsequently was fixed in a TSF on November 4, 2014, for gradual correction and he achieved uneventful healing after 184 days with both legs even in length.

**Patient 13** is a 31-year-old otherwise healthy man who sustained an open distal tibial fracture in a motor vehicle accident at the age of 7 years. The fracture healed with a severe s-shaped 20^o^ varus deformity and 70 mm shortening. The skin was adherent to the distal-medial part of the tibia and ankle. On February 6, 2014, he underwent a corrective osteotomy at the fracture site and a proximal osteotomy for lengthening. The distal osteotomy could not correct the deformity completely owing to the skin condition. The lengthening is now completed and the frame was removed on May 16, 2014, after 99 days.

**Patient 14** is a 21-year-old woman from Gambia with multiple cartilaginous exostoses, genu valgum of 30^o^, and valgus deformity of the distal tibia of 20^o^. On February 3, 2014, she underwent an acute correction of the left distal tibia, and resection of exostoses of her left distal femur and proximal tibia and fibula. During the same surgery we released the peroneal nerve and performed a proximal tibial osteotomy for gradual correction of the valgus deformity. Both osteotomies were fixed in a TSF and have now healed uneventfully. The frame was removed May 28, 2014, after 114 days.

**Patient 15** is a 52-year-old man who presented with a pseudarthrosis which was fixed with a nail on January 14, 2014. On February 6, 2014, the nail was removed owing to infection, and he underwent application of an external Hoffmann frame with cement and antibiotics. On May 12, 2014, the Hoffmann frame was replaced by a TSF with removal of the cement, and he had an osteotomy in the proximal tibia. On July 30, 2014, he underwent refreshment of the docking site and bone grafting without removal of the TSF. His progress was slow, therefore it was decided after his second PET/CT scan to use ultrasound stimulation to encourage bone growth. He had a third PET/CT after another 6 weeks. He eventually achieved healing, and the TSF was removed on October 12, 2015, after 518 days.

**Patient 16** is a 40-year-old otherwise healthy man who sustained an open proximal tibial fracture in April 2013. He was treated with an acute temporary external fixator (Hoffmann II^TM^), and then underwent percutaneous plating on April 17, 2013. He was randomized to receive hyperbaric oxygen treatment in a randomized controlled study. October 29, 2013, he still experienced pain and radiologically there was a nonunion and varus and procurvatum deformities of 10^o^ each. On March 13, 2014, he underwent a proximal tibial osteotomy and fixation with a TSF circular frame for gradual correction of the deformity. He achieved uneventful healing and the frame was removed August 12, 2014, after 152 days. He now is fully weightbearing, with no pain. He had one PET scan before and the second after removal of the TSF because of a scheduling delay.

**Patient 17** is a 65-year-old man with high blood pressure but otherwise is healthy. On July 2014 he fell 2 m from a ladder and sustained a comminuted intraarticular open distal tibial “pilon tibiale” fracture. He had temporary stabilization of the fracture in a Hoffmann II^TM^ external fixator, which was exchanged for a TSF circular frame on July 21, 2014. A plain radiograph showed callus formation. He is now fully weightbearing with no pain or infection. He healed uneventfully and the frame was removed on December 15, 2014, after 147 days.

**Patient 18** is a 29-year-old otherwise healthy construction worker who fell 8 m on July 22, 2014. He sustained an unstable L2 vertebral fracture with loss of sensory and motor function below his right knee, a wrist fracture, and a comminuted intraarticular open distal tibial “pilon tibiale” fracture. The tibial fracture was immediately reduced with application of a Hoffmann II^TM^ external fixator. The vertebral fracture was fixed with rods the same day, after which he gradually regained function in his right leg. The wrist fracture was reduced and held in a cast until July 27 when it was fixed with a plate and screws. On July 31, the external fixator was extracted and replaced by a TSF circular frame. The patient gradually started to bear weight and was able to terminate taking pain medication. A plain radiograph shows healing of the fracture is progressing. The frame was removed on February 15, 2015, after 199 days.

**Patient 19** is a 65-year-old man who sustained a wound from a chainsaw to the distal third of his left tibia in January 2013. The wound was irrigated and revised but shortly after this it became evident that a fissure in the tibia had been missed as the fracture dislocated and therefore, was subsequently fixed with a plate and screws. A deep infection and malunion developed and in December 2013 he underwent removal of the plate that also by then was broken. After revision surgery there was a 2-cm bone defect that was filled with a cement spacer with gentamicin. The tibia was now stabilized with a hybrid external fixator. In February 2014 the cement spacer was removed and the bone defect was filled with autologous bone graft. However, the infection was still present and the bone graft therefore was reabsorbed. The patient was referred to us and on October 23, 2014, he underwent resection of all dead bone and a proximal osteotomy for a subsequent bone transport in a TSF. The bone transport was uneventful but the callus formation was slow leading us to perform autologous bone grafting to the regenerate at the distraction osteotomy site and percutaneous drilling of the docking site. This was done in May 2015. He then achieved bony union and the infection resolved. The frame was removed November 13, 2015, after 386 days.

**Patient 20** is a 59-year-old man with monoarthritis in his left ankle. For this reason he had two surgeries—one in 2008, and in March 2014, he underwent a subtalar arthrodesis. After this he had a fulminant infection that was drained in Italy where he spent his summer vacation. On August 14 he presented to our emergency ward at Karolinska University Hospital and 2 days later he underwent a revision surgery and removal of all hardware. Four days later he was stabilized in an external fixator and vacuum-assisted closure (VAC) was initiated. After changing the VAC a few times he again underwent surgery on September 19, 2014, when the bone defect was revised and filled with a cement spacer with gentamicin. On November 5, 2014, the Hoffmann-type external fixator was changed to a TSF. We removed the cement spacer and filled the defect with a mixture of autologous bone graft and calcium sulphate mixed with tobramycin and vancomycin. After a minor operation because of a pin infection in the forefoot the frame was removed on April 27, 2015, after173 days. The infection has now subsided and he has a stiff pain-free ankle. He is due for a radiograph within 6 months to confirm complete consolidation.

**Patient 21** was a 45-year-old man with obesity, high blood pressure, high blood fats, posttraumatic stress syndrome, and depression owing to the recent loss of his wife and sister. He was admitted to the emergency/trauma room on June 10, 2014, after an assault where he sustained an open fracture of the distal tibia and fibula (Gustilo-Anderson II). He was immediately taken to the operating room for débridement and fixation with a Hoffmann external fixator. The wound was sutured and after recovery of the skin and soft tissues the external fixator was removed and the fracture fixed with minimally invasive plate osteosynthesis on June 27, 2014. Postoperatively he was treated with oral antibiotics (flucloxacillin and later clindamycin) because of discharge from the wound. After removal of antibiotics he again was admitted on September 1, 2014, for débridement owing to fever, pain in the leg, local severe swelling, redness, and discharge. After reoperation he again was treated with oral antibiotics. Owing to persistent discharge and later breakage of the plate and screws, he underwent surgery January 15, 2015, where all metal was removed, bacterial culture samples were obtained, the infected pseudarthrosis was filled with calcium sulphate mixed with gentamicin, and the pseudarthrosis was stabilized with a TSF. Owing to lack of appropriate stability a foot plate was added to the frame on January 31, 2015. The patient received one PET scan but then was lost to followup, as he was found deceased in his home 57 days after the TSF was attached.

**Patient 22** is a 78-year-old woman with high blood pressure and insulin-dependent diabetes mellitus for several decades. She was admitted to the emergency room January 21, 2015, after slipping on ice where she sustained an open fracture of the distal tibia and fibula (Gustilo-Anderson I). She initially was treated with external fixation with a Hoffmann fixator. Owing to the nature of the fracture (open fracture), soft tissue conditions, and comorbidity (diabetes mellitus), the initial external fixator was changed to a TSF on January 29, 2015, to improve stability and enable weightbearing. Soft tissues healed uneventful. The fracture healed and the frame was removed June 17, 2015, after 139 days.

**Patient 23** is a 24-year-old medical student who experienced an open comminuted pilon fracture of the distal tibia and a nondisplaced calcaneal fracture on April 16, 2014. The pilon fracture was treated elsewhere and the patient was left with an unclosed wound anteriorly because of skin tension. When the patient returned to Sweden in May, the wound was covered with a partial skin graft after treatment with VAC. The skin recovered but pseudarthrosis (nonunion) in the distal tibia and posttraumatic osteoarthritis in the ankle developed. On February 9, 2015, the patient underwent removal of the plate and screws, autologous bone grafting, and Stimulan® (CaSO4) (Biocomposites, Ltd, Keele, Staffordshire, England) mixed with vancomycin. The fracture was fixed in a TSF and the ankle was distracted (arthrodiastasis) to improve the pain from the arthritis. The nonunion healed and the frame was removed May 18, 2015, after 98 days. The patient still has ankle pain owing to osteoarthritis but the fracture is radiologically united.

**Patient 24** is an otherwise healthy man who in March 2012 was mountain climbing in Turkey and fell, sustaining an open comminuted fibular fracture. He was transported by helicopter to a hospital where the wound was cleaned and the fracture was stabilized with an external fixator. On returning to Sweden the external fixator was removed and the fracture reduced and fixed with a plate and screws on April 2, 2012. Two months later he was due for removal of a syndesmosis screw. Owing to suspicion of delayed union the screw was left in place. One month later the patient returned with a fracture dislocation and a subluxed ankle. For some reason there now also was a pilon fracture that either earlier was nondisplaced or was caused by intensive mobilization of this highly motivated patient. The fractures were again fixed with plates and screws. He then underwent revision surgery twice because of infection, and on May 6, 2013, he underwent removal of plates and screws, an arthrodesis with distal fibula as an autologous bone graft, and fixation in a TSF. The patient was fully weightbearing and the frame was removed July 25, 2013. The patient wore a cast for 1 month and then returned to normal activity. Unfortunately the arthrodesis gradually displaced into almost 30^o^ valgus and because of this he again underwent surgery on March 15, 2015. We did a closing wedge osteotomy and mixed the wedge with Stimulan® mixed with vancomycin. The osteotomy was again fixed with a TSF, which was removed August 18, 2015, after 156 days. The patient has a pain-free arthrodesis, is very active, and walks in the forest during hunting seasons.
